# A mixed methods exploratory evaluation of burnout in frontline staff implementing dialectical behavior therapy on a pediatric eating disorders unit

**DOI:** 10.1186/s40337-021-00453-1

**Published:** 2021-08-13

**Authors:** Jennifer Couturier, Zechen Ma, Liah Rahman, Cheryl Webb

**Affiliations:** 1 Department of Psychiatry and Behavioural Neurosciences, McMaster University, Hamilton, ON Canada; 2 Faculty of Health Sciences, McMaster University, Hamilton, ON Canada; 3 McMaster Children’s Hospital – Hamilton Health Sciences, 1200 Main Street W, Hamilton, ON L8N 3Z5 Canada

**Keywords:** Dialectical behavior therapy, Burnout, Eating disorders, Nursing staff, Implementation

## Abstract

**Background:**

Eating disorders are life-threatening illnesses that commonly affect adolescents. The treatment of individuals with eating disorders can involve slow treatment progression and addressing comorbidities which can contribute to staff burnout. Dialectical behavior therapy (DBT) has emerged as a viable treatment option and has reduced staff burnout in several other settings. Our aim was to describe frontline staff burnout using mixed methodology on a DBT-trained combined inpatient/day hospital unit for pediatric eating disorders.

**Method:**

Frontline staff were trained to provide DBT skills for adolescents with eating disorders. Twelve months following the training and implementation, they completed the Copenhagen Burnout Inventory (CBI) and a qualitative interview. Directed and summative content analyses were used.

**Results:**

Eleven frontline staff including nurses, child life specialists and child and youth workers participated. The CBI revealed that only one staff member experienced high personal burnout, while another experienced high client-related burnout. Qualitative data indicated that all frontline staff felt DBT had the potential to reduce burnout.

**Conclusion:**

Qualitative data indicate that staff believe that DBT may hold promise in reducing burnout for pediatric frontline staff who treat children and adolescents with eating disorders. Further study is needed.

**Plain English summary:**

Understanding burnout is particularly important for nursing staff in inpatient and day hospital settings for eating disorders, as nursing staff generally have the most frequent patient contact; thought to be a risk factor for burnout. The reduction of burnout can prevent detrimental effects on job performance, personal well-being, and patient outcomes. Our exploratory study shows that frontline staff believe that DBT may have the potential to reduce burnout in staff treating children and adolescents with eating disorders in a combined inpatient/day hospital setting. Further study is needed in this area.

## Introduction

Eating disorders (ED) are the third most common chronic illness that affect adolescents [1]. These illnesses can be life threatening, with anorexia nervosa having the highest mortality rate of any psychiatric illness [2]. Not only do ED affect the sufferers’ physical, emotional, and social lives, but also those of their families [3, 4]. The treatment of ED presents unique challenges to treatment providers for many reasons. Patients with ED frequently experience comorbid psychiatric diagnoses such as depression, anxiety, obsessive–compulsive disorder, personality disorders, and substance abuse disorders [3, 5]. Additionally, behaviors such as self-harm in this group of patients are perceived as very stressful to treatment providers [6, 7]. Slow progress in treatment, chronicity, relapse, and symptom severity also make treatment providers particularly prone to burnout [7, 8]; a syndrome of emotional exhaustion, depersonalization, and a lack of sense of accomplishment [9, 10].

Frontline mental health care providers are thought to be particularly at risk for burnout given the nature of the work which includes high workload, demanding caregiving relationships with patients, secondary traumatization, and dealing with suicide and self-harm [9, 10]. A qualitative study involving 298 ED treatment providers examined primary contributors to burnout and found the following themes: (1) the nature of the ED including treatment resistance, (2) patient characteristics such as comorbidity, (3) work-related factors such as lack of support, high work demands, (4) therapist variables including negative affect and countertransference, and (5) financial issues such as inadequate payment [8]. Ninety-three per cent of providers said that they worried about their patients’ health, and about 20% had experienced the death of a patient resulting in feelings of sadness, self-doubt, guilt and helplessness. It is important to note that participants in this study were recruited through membership in various eating disorder organizations (the Academy for Eating Disorders), treatment centres and listservs, and thus, did not specify whether they were pediatric or adult service providers. Patient death may not be as commonly experienced by pediatric service providers, although it does occur. Staff also mentioned their efforts to avoid burnout including: self-care (exercise, social supports and hobbies), professional support (consultation, supervision), limiting caseload, personal therapy, professional activities, spirituality/religion and maintaining a sense of humor [8]. An additional study reporting on this same sample of ED treatment providers found that job burnout was associated with being younger, female, overweight, working longer hours, having less experience, and experiencing a patient death [11].

Although fewer studies have been conducted in the pediatric ED realm with respect to burnout, clinician attitudes toward adolescents with ED have been documented [12, 13]. One study involving 120 clinicians treating adolescents with ED reported six patterns of reactions including angry/frustrated, warm/competent, aggressive/sexual, failing/incompetent, bored/angry at parents and overinvested/worried [13]. An additional qualitative study examining attitudes and perceptions of physicians, registered nurses, and care assistants treating adolescents with ED on a general pediatric unit found several themes including awkwardness and uncertainties of care, navigating family dynamics, and establishing therapeutic boundaries, as challenging [12].

Taken together, these findings from previous research suggest that clinicians can have complex responses when working with patients with eating disorders such as anger, anxiety, and helplessness, suggesting that frontline workers may be at increased risk of burnout. Given these perceptions, and risks of job burnout in the field of ED, it is important to better understand this phenomenon in order to ascertain potential mitigating strategies to avoid burnout.

Although Family-Based Treatment (FBT) is the treatment of choice for adolescents with eating disorders [14, 15], a significant proportion of young people do not recover with this treatment alone [16]. From previous research we know that treatment resistance can increase feelings of burnout [8]. Given this, in combination with the context of high levels of burnout in the field of ED by treatment providers, the investigation of novel treatments that could be applied in the field of pediatric ED as adjuncts to FBT with possible added benefit to staff would be prudent. Dialectical behavior therapy (DBT), an intervention that integrates mindfulness and acceptance strategies with traditional behavior therapy, has emerged as a viable intervention for adults with ED [17–19]. The treatment originated from caring for patients with borderline personality disorder (BPD) and has been found to be successful in reducing a range of problems such as suicidal ideation and attempt, self-injury, depression, and substance abuse [17, 20]. In a systematic review on the efficacy of DBT for bulimia nervosa, binge eating disorder, and anorexia nervosa, DBT was able to reduce the frequency of ED behaviors in adults and adolescents [19]. In addition, the value of adding DBT as an adjunct to FBT models for adolescents has recently been emerging [21, 22].

With respect to its potential impact on burnout, further research suggests that mental health staff in a pediatric residential treatment facility who received DBT-informed training, reported lower personal and work-related burnout [23]. And in another treatment setting, when clinicians were trained in DBT to help young women with BPD symptoms, learning DBT was seen as stressful in terms of demands on time and energy, however, its mindfulness training facet decreased the experience of stress in the treatment of patients [6]. Thus, DBT may have the potential to reduce stress and burnout in staff and may have benefits as an adjunctive treatment for young people with ED.

Research on DBT implementation to date has focused on masters-level clinicians and mental health professionals treating adults and adolescents with BPD [6, 23], while its effects on nursing staff treating children and adolescents with ED are unexplored. Understanding burnout is particularly important for nursing staff in ED inpatient and day hospital settings, as nursing staff generally have the most frequent patient contact, high work load, and work long hours; all risk factors associated with burnout in ED treatment providers [11]. As documented in other studies, the reduction of burnout can prevent detrimental effects on job performance, personal well-being, and patient outcomes [24]. Thus, the current study aims to examine burnout in an exploratory fashion in frontline staff working in a child and adolescent combined inpatient and day hospital ED setting one-year post-DBT training and implementation.

## Methods

### Design

This is a cross sectional study examining quantitative and qualitative outcomes one year following DBT training for frontline staff on a combined inpatient/day hospital ED unit. This study was reviewed and approved by the Hamilton Health Sciences/McMaster Faculty of Health Sciences Research Ethics Board. The one-year period was chosen in order to ensure that DBT was fully adopted into the program which required a culture shift, and time.

### Participants

A purposeful sample of registered nurses, child life specialists (a university degree speciality program focused on the developmental impact of illness and injury), and child and youth workers (college level trained individuals specializing in child behaviour) currently working on a combined inpatient/day hospital ED unit were invited via email by co-author CW to participate in this study investigating DBT implementation. The frontline staff work alongside a team of pediatricians, psychiatrists, psychologists, social workers, a dietician, and doctors-in-training on a combined inpatient and day hospital unit for children and adolescents with ED.

Of 26 eligible frontline staff members who are employed on the unit, 11 agreed to study participation and completed the Copenhagen Burnout Inventory (CBI) and a qualitative interview. Participants all identified as female and ranged in age from 25 to 55 years. There were 9 nurses, 1 child life specialist, and 1 child and youth worker. The participants had at least one-year experience working in their profession and the majority of participants (91%) had more than four years of experience working with pediatric patients with ED. A mix of occasional (n = 2), part-time (n = 4), and full-time (n = 5) staff were recruited. In terms of their experience with DBT training, all reported that they had received the full formal training provided by our program.

### Setting

The inpatient unit mainly admits those under 18 years for medical reasons and consists of six beds, while the day hospital program is physically located in the same space, and consists of four patients who attend five days a week and sleep at home. It is important to note that our program has a main theoretical orientation of FBT, preparing parents to take on the role of nutritional rehabilitation at home, upon discharge. However, DBT, particularly skill use, was implemented to be used in combination with these principles in order to reduce patient distress associated with re-nourishment.

Those inpatients who are medically stable enough to participate in group are combined with the day hospital patients in a daily DBT skills group. Bedside DBT skills are taught to those who are not medically stable enough for group. A description of patient characteristics and program content in our day hospital program has previously been published [25]. The program consists of school in the morning and groups in the afternoon for those who are medically stable. A one-hour formal DBT skills group is held daily and a distraction group (consisting of art, music, games or puzzles) largely related to the skill taught that day is also held for an hour in the afternoon. All meals and snacks are closely supervised. Those patients who are in the DBT stream also complete diary cards and chain analysis, whereas those who are in the FBT stream might only attend groups.

### Training

Formal DBT skills training was provided for a 6-month period by a DBT expert for all staff (including MDs). Staff were trained through several formal training days and were provided with manuals, videos and online learning modules on DBT. Following the 6-month training, staff began practicing DBT for patients through group co-facilitation, meal support, and bed-side care in our combined day hospital and inpatient setting. Consultation groups for staff were not utilized due to shift work and constraints on time. However, daily medical rounds were attended by all staff (including pediatricians, therapists, and the psychiatrist) and often included focus on DBT skill use for patients. Staff who were not able to fully participate in training due to demands of the ward or leaves of absences were supplemented with half day workshops offered twice in the year, as well as one-on-one teaching (these individuals were not included in our analysis).

### Measures

Demographic data were collected on profession, number of years of experience working in the profession, number of years of experience working with pediatric ED population, type of contract (full time, part-time, occasional), and experience with DBT.

The Copenhagen Burnout Inventory (CBI) [26] was used to assess burnout 12 months following the initiation of training and implementation of DBT. The CBI was administered along with a demographic data collection sheet, and a semi-structured qualitative interview developed by the authors on the uptake of DBT and conducted by a research assistant (LR).

The CBI is a nineteen item questionnaire used to assess three different subscales of burnout including: (1) Personal burnout: related to physical and psychological exhaustion (six items); (2) Work-related burnout: related to aspects of the work environment (seven items); and (3) Client-related burnout: related specifically to interacting with clients (six items) [26]. Each item is scored on a 5-point Likert scale with options being 0 (never/almost never), 25 (seldom), 50 (sometimes), 75 (often), and 100 (always). Scores on the subscales are an average of the items contained within the subscale and range from 0 to 100, with higher scores representing a greater degree of burnout [26]. High degree of burnout is defined as a score of 50 points or higher on the subscale score. The CBI has shown good reliability and criterion-related validity in a previous study conducted on 1,914 Danish service employees including midwives, social workers, nurses, and prison ward staff [26, 27].

### Qualitative procedures

The principles of fundamental qualitative description [28] guided sampling, data collection, and analytic decisions in this study. This qualitative approach is appropriate for identifying the fundamental structures of a phenomenon, including an exploration of individuals’ experiences and perceptions, and then providing a comprehensive summary of events. Sandelowski [28] identifies that this design is particularly useful for identifying practical and relevant knowledge to address clinical and policy challenges experienced by different levels of decision-makers. An in-depth semi-structured qualitative interview was completed with each participant, which was focused on the experiences of frontline staff implementing DBT. Staff were asked about their experiences of DBT and whether they felt DBT had the potential to reduce burnout (why or why not?). All interviews were recorded, transcribed verbatim and analyzed for themes.

### Analyses

#### Quantitative

The mean scores and standard deviations for each subscale of burnout were calculated. Software SPSS version 19.0 was used to examine our quantitative data.

#### Qualitative

Qualitative content analysis (including directed and summative approaches) [29] was used to examine our transcripts for themes. A directive/deductive analysis was used as we began our analysis with a theoretical framework in mind, in alignment with the CBI major categories of Personal, Client-Related and Work-Related burnout. If participants mentioned the application of DBT skills in their personal lives and how this relates to burnout, this was coded under “Personal”. If they mentioned skill use with respect to clients/patients with respect to burnout, this was coded under “Client-Related” and if they discussed application of skills at work in a general fashion (i.e. On the unit, or in the workplace) related to burnout, this was placed under “Work-Related”. Summative content analysis was used to count the number of times subthemes were mentioned within each category of our theoretical framework. First, all transcripts were read in their entirety to achieve an overall understanding of the scope of the data. Then, one of the authors (CW) conducted line-by-line coding to generate an initial list of codes. A codebook with definitions of each code and theme was generated and refined through multiple readings of the transcripts, in consultation with the research team. As new codes and themes emerged from the transcripts, they were added to the codebook and documented in the research audit trail. The collapsing of codes into themes and subthemes was documented in the same fashion. To ensure the dependability of the data analysis, 20% of the transcripts also were independently coded by co-author (JC). There was a process of discussion in place to arrive at consensus on codes. The initial definitions of the codes and themes were reviewed and references of that code or theme were discussed in relation to the initial definition (there were no disagreements). Saturation was achieved by the eleventh transcript. All coding was completed in Nvivo 12, a software program used to organize and store qualitative data for synthesis.

## Results

### Quantitative results

In terms of the CBI, staff experienced an average personal burnout score of 35.2 (SD 11.8, range 12.5–50.0), an average work-related burnout score of 31.5 (SD 10.6, range 10.7–42.9), and an average client-related burnout score of 26.5 (SD 15.6, range 4.2–58.3). One staff member experienced high personal burnout and another staff member experienced high client-related burnout. None of the participants experienced high overall burnout.

### Qualitative results

See Table 1 for a description of themes and subthemes arising from our data. All participants indicated that they felt DBT had the potential to reduce burnout and compassion fatigue. Nine out of 11 participants also raised some concerns about the implementation of DBT and how this could have the potential to increase stress and burnout.

**Table 1:** Themes and subthemes emerging from qualitative analysis (n = 11 transcripts)

Theme	Subtheme	Frequency
Personal	Using skills in everyday life Most helpful skills Uncertainty	7 participants, 10 references 4 participants, 4 references 3 participants, 5 references
Work-Related	Feel more effective My job is easier Validate/support each other Competing demands	6 participants, 8 references 6 participants, 7 references 2 participants, 4 references 2 participants, 5 references
Client-related	Reduces anxiety of client Not suitable for some clients Frustrating if clients refuse	3 participants, 4 references 3 participants, 5 references 1 participant, 2 references

#### Personal burnout

Subthemes which arose in the personal burnout category included: (1) Using Skills in Everyday Life, (2) Most Helpful Skills, and, (3) Uncertainty.

*Using skills in everyday life*—Several front-line staff mentioned that using skills in everyday life is a form of self-care, and is helpful to them personally. They mentioned things they do in terms of going to the spa or taking time for oneself in relation to DBT philosophy. They discussed that whether you are struggling with a specific issue such as an eating disorder, or stress related to work, that skills can be equally applicable. One staff mentioned this with respect to using skills in their everyday life,

Yeah, definitely. I see myself definitely using them. So yeah, I think that they do (reduce burnout), and I don’t think it just needs to be for our program. I think it’s just the skills people need to use in general, like in their everyday lives more. P5

*Most helpful skills—*Frontline staff mentioned mindfulness, self-soothe, distraction, and interpersonal effectiveness as the most helpful skills that they use in their everyday lives. They mentioned that their relationships have been improved when they use interpersonal effectiveness skills and that mindfulness helps with burnout. One frontline staff said this about mindfulness,

Yes. Especially if you’re using the skills with your own self, you’re taking that time for self-care….. Oh, with the mindfulness piece, being in the moment and even driving to and from work, taking the time to de-stress and breathing, I think those would the biggest things and just the awareness that comes with the DBT and having it as part of the day- to-day life. P13

*Uncertainty*—Uncertainty about whether DBT could reduce burnout personally was also expressed by several participants. Some felt that they did not use DBT skills religiously, or frequently enough, and thus, they might not be as effective. There was also a suggestion that buy-in with respect to the DBT model was important in helping with personal burnout. One participant expressed her uncertainty in this fashion,

Oh, I don’t know if it would speak to burnout, like having less burnout. I mean I didn’t work in the field long enough beforehand to maybe know what it felt like, so I don’t know that I can really truthfully answer that. P6

#### Work-related burnout

Subthemes in the work-related burnout category included: (1) Feel more Effective, (2) My Job is Easier, (3) Validate/Support Each Other, and (4) Competing Demands.

*Feel more effective—*Several participants mentioned that training in DBT skill use has decreased feelings of helplessness and made them feel more effective in their jobs. They discussed the feeling of “breaking through” to a client with a skill and how that helps them to feel competent in their role as a frontline staff. With respect to feeling more effective, one frontline staff had this to say,

Yes, I do. I do, because I think the feeling of helplessness, of not knowing how to help a child is what causes the burnout, a lot of it. So having the DBT, that’s given us really specific guidelines on how to help with skills. I think then you’re more effective, and when you’re more effective, I think you reduce the burnout because you want to be effective…. P4

*My job is easier*—Participants mentioned the consistency and predictability on the unit which has been enhanced with DBT skill use and module teaching. Some mentioned the unit runs more smoothly as clients can regulate themselves better with skill use. One participant said this about the unit,

Yeah, I think it’s just more consistency and more predictability, knowing what we’re doing all day and knowing that, “Okay, this entire week, we’re working on this skill”. P10

*Validate/support each other*—Two staff mentioned that DBT language helps them to validate each other in a supportive way, and that skill implementation has provided an opportunity to enhance validation skills for each other on the unit. They mentioned that use of validation skills amongst the whole team creates a more supportive work environment. One staff mentioned this with respect to validation,

Yeah, I guess it does. I've never actually thought of it in that way before, but I think so because, yeah, we can support each other more. So, staff member to staff member, we can be more validating in what we say and help each other out that way. P1

*Competing demands*—Frontline staff mentioned the difficultly they face in managing all the tasks they must do on a daily basis including managing the medical safety of patients, along with learning and teaching DBT skills. They reported they often feel they do not have enough time to devote to bedside care of patients, in addition to running/participating in DBT group, and teaching skills individually to patients. They reported that these competing demands can increase burnout. One participant had this to say in terms of of time,

In that sense, I think it can cause fatigue, burnout in nurses if they’re expected to do all these things that they don’t really have the time to do. But otherwise I think it’s good. P7

## Client-related burnout

With respect to client-related burnout, subthemes included: (1) Reduce anxiety of client, (2) Not Suitable for Some, and (3) Frustration.

*Reduce anxiety of client*—Several participants mentioned that DBT skills are effective for reducing anxiety of the patient and helping them regulate their emotions. Participants also mentioned that the validation skills can be helpful in de-escalating high emotional situations with family members as well. One participant said this regarding reducing the anxiety of a client,

It can definitely help in reducing a high emotional situation or something with a patient if they're able to use DBT skills in the moment. Because then that also helps with reducing their anxiety, their stress, and that then helps us, too, obviously. Then, I think that would help the burnout, too. P3

*Not suitable for some patients—*Participants mentioned that many patients are not suitable for DBT skills. These patients might be medically unstable or unwilling to engage in skill use. A trend of increasing patient complexity was mentioned by frontline staff and how this can increase stress and burnout. One participant mentioned that inpatients might be too malnourished to comprehend or focus on skills. One frontline staff had this to say regarding client suitability,

But like I say, I feel like it works generally better with the day treatment patients, because they’re just more willing to learn about it. I think with the inpatients, it’s harder. We’re just trying to get them stable sometimes, like medically, and their brain is too starved to register what you’re talking about when you’re talking about all these skills. P7

*Frustration*—Staff mentioned that it can be very frustrating to try to teach patients skills who really do not want to learn them. They expressed that this could increase burnout. One frontline staff had this to say about feeling frustrated when clients refuse to learn skills,

It can also be extremely infuriating because kids, some of our patients often get very frustrated with the idea of having to learn skills. And again, I think it’s primarily because, even if they don’t see it, they’re just not in a place where they want to get better, so why would they want to learn skills to fix the problem that they don’t really want gone? P5

In summary, our qualitative data generally suggest that frontline staff feel that the use of DBT skills can reduce burnout in personal, work-related and client-related areas, although they also expressed issues of frustration, competing demands and time pressures.

## Discussion

This is the first study to use a mixed methods exploratory approach in evaluating burnout one year following DBT implementation in frontline staff on a child and adolescent ED unit. Our quantitative data in the form of mean scores on the CBI indicated that although none of the staff were experiencing high overall burnout, one staff reported high personal burnout and one staff reported high client-related burnout. Our qualitative results also support the use of DBT in helping frontline staff with burnout in personal, work-related and client-related realms. All eleven participants mentioned the potential for DBT to reduce burnout, and the majority (7/11) focused on personal burnout in their interviews, reporting that skills help them in their everyday lives. Frontline staff commented on their own use of mindfulness skills in their personal lives, the support of each other in terms of validating each other and learning skills together in terms of work-related burnout, and feeling effective instead of helpless when dealing with clients. They also mentioned the stress involved in learning a new treatment modality which can contribute to burnout. In addition, they discussed not having enough time to learn and teach DBT skills. They mentioned competing demands in that they are looking after medically unwell children and often do not have the time to sit at the bedside to teach skills. They also mentioned that not all clients are suitable, and that it is frustrating when clients refuse to use skills. And, finally, they mentioned that there must be buy-in with respect to the treatment in order to adopt it, with some expressing uncertainty that DBT can reduce burnout.

The key components of DBT in terms of the benefits for frontline staff in reducing burnout are yet to be fully elucidated. The absence of consultation groups in both our sample and in the study by Haynos and colleagues [23] indicates that this component of DBT implementation for frontline staff may not be essential, and that other components of DBT may contribute to burnout reduction, although further study is needed. Other studies have suggested that reductions in burnout may be attributed to DBT’s systematic incorporation of common coping strategies used by mental health nurses such as stress management that involves social support, limit recognition, skill improvement, and peer support that are reported to be helpful in coping with stress [6, 17, 30]. Additionally, the reduction in burnout may also be related to DBT’s mindfulness component involving meditation, relaxation, and acceptance techniques for both the care provider and patients [17]. In previous studies, mindfulness skills have been expressed by therapists treating patients with BPD as most helpful [6], while in community and university settings, healthcare providers experienced burnout reductions and improved mental well-being after mindfulness-based stress reduction interventions [31]. Yoga-based stress management and cognitive behavioural stress management strategies have both been found to be helpful in improving physical and mental health of frontline mental health care providers; both having mindfulness components [32]. Similarly, mindfulness was reported to be helpful for stress reduction in our sample of frontline staff. In addition, peer support in terms of validation and skill use were mentioned repeatedly in the qualitative interviews of frontline staff in our study.

One limitation of the current study is the low study participation rate from staff on the unit. One potential bias is that staff with high levels of burnout may have felt too exhausted to participate. As the CBI was used in addition to a semi-structured interview, this may have added a time barrier that discouraged participation. We do not have data available on staff who did not participate to know whether they were full-time, part-time, or casual employees, or whether they were currently on a leave of absence such as a maternity leave. Additionally, other factors that may also increase employee burnout such as other negative life events were not captured [33]. As some staff members have less than one year of experience with DBT, their burnout scores may be higher, as DBT is seen as stressful due to high learning demands during the first 6 months in other studies [6]. Additionally, there was some variability in the uptake of DBT training within the staff due to work demands and leaves of absences. With a larger sample size, it would be meaningful to compare level of experience in providing DBT and level of burnout. In addition, burnout was not measured pre-DBT training to allow for comparison of burnout between pre- and post-DBT implementation. Saturation of our qualitative data occurred at the eleventh transcript. It is possible that more transcripts would result in more themes that were not captured in our sample.

## Conclusions

Our exploratory quantitative and qualitative findings suggest that training frontline clinicians in DBT skills may have benefits. Frontline staff reported that there is potential for DBT training to have an impact on burnout, indicating that further study is needed. Future investigations into staff burnout in pediatric eating disorders settings could consider using the CBI as an instrument to quantify burnout, and should consider further qualitative research to investigate the complex nature of burnout when implementing a new treatment. Further investigation is warranted on the experience of the care team and administrators to identify facilitators and challenges to implementing DBT in a pediatric ED setting.

## Data Availability

The datasets used during and/or analysed during the current study are available from the corresponding author on reasonable request.

## Abbreviations

BPD: Borderline personality disorder
CBI: Copenhagen burnout inventory
DBT: Dialectical behaviour therapy
ED: Eating disorder
